# Wnt5a promotes migration of human osteosarcoma cells by triggering a phosphatidylinositol-3 kinase/Akt signals

**DOI:** 10.1186/1475-2867-14-15

**Published:** 2014-02-14

**Authors:** Ailiang Zhang, Shuanghua He, Xiaoliang Sun, Lianghua Ding, Xinnan Bao, Neng Wang

**Affiliations:** 1 Department of Orthopaedics, Changzhou No. 1 People’s Hospital, Changzhou, Jiangsu 213003, PR China

**Keywords:** Wnt5a, PI3K, Akt, Osteosarcoma, Migration

## Abstract

Wnt5a is classified as a non-transforming Wnt family member and plays complicated roles in oncogenesis and cancer metastasis. However, Wnt5a signaling in osteosarcoma progression remains poorly defined. In this study, we found that Wnt5a stimulated the migration of human osteosarcoma cells (MG-63), with the maximal effect at 100 ng/ml, via enhancing phosphorylation of phosphatidylinositol-3 kinase (PI3K)/Akt. PI3K and Akt showed visible signs of basal phosphorylation and elevated phosphorylation at 15 min after stimulation with Wnt5a. Pharmaceutical inhibition of PI3K with LY294002 significantly blocked the Wnt5a-induced activation of Akt (p-Ser473) and decreased Wnt5a-induced cell migration. Akt siRNA remarkably inhibited Wnt5a-induced cell migration. Additionally, Wnt5a does not alter the total expression and phosphorylation of β-catenin in MG-63 cells. Taken together, we demonstrated for the first time that Wnt5a promoted osteosarcoma cell migration via the PI3K/Akt signaling pathway. These findings could provide a rationale for designing new therapy targeting osteosarcoma metastasis.

## Introduction

Osteosarcoma, characterized by a high malignant and metastatic potential, principally affects children and adolescents [1]. Progression of disease is inexorable and response to therapy can be unrewarding: fewer than 50% of patients live beyond 10 years, and there are no reliable predictors to guide the choice or intensity of therapy [1,2]. Several improvements in understanding the molecular pathology of metastatic osteosarcoma have been achieved in the last several years [1]. However, the molecular mechanisms underlying this malignancy are still largely unknown. For this reason, elucidating the signaling pathways involved in the metastatic cascade has become a key goal for developing novel effective therapeutics aimed at reducing osteosarcoma mortality rates.

Wnt signaling regulates several developmental and oncogenic processes in both insects and vertebrates [3]. Signals triggered by Wnt are classified into two groups, a canonical β-catenin pathway and non-canonical pathways. In the canonical pathway, Wnt signals are mediated by disheveled (Dvl), which inhibits glycogen synthase kinase-3β (GSK-3β) activity [4]. The accumulated β-catenin enters the nucleus, forms complexes as a co-factor with Tcf/Lef transcription factors, and then triggers transcription of a set of target genes, which ultimately leads to regulation of cell proliferation and cell fate as well as cell transformation [5]. Compared to the canonical pathway, the non-canonical Wnt pathways are not fully understood, especially in mammals, while its contributions to cell polarity in *Drosophila* (a planar cell polarity pathway, a PCP pathway) and convergent extension movement in *Xenopus* and zebrafish are demonstrated [6-9]. Wnt5a has been originally classified into the non-canonical Wnt group and reported to trigger Ca^2+^ pathways that subsequently activate protein kinase C (PKC) and Ca^2+^/Calmodulin kinase II [10,11]. Wnt5a can activate other intracellular protein kinases such as extracellular signal-regulated kinase (ERK), Akt and c-Jun N-terminal kinase (JNK) [12,13].

It has been reported that trimeric G protein mediated activation of phospholipase C and phosphodiesterase is involved in Wnt-induced gene expression [14]. Phosphatidylinositol-3 kinases (PI3Ks) are a family of enzymes involved in cellular functions including cell growth, proliferation, differentiation, motility, survival and intracellular trafficking, all of which can potentially influence the development of cancer. The serine/threonine kinase Akt is a major effector of the PI3K pathway and is activated by many polypeptide growth factors [15]. In this study, we hypothesized that the PI3K/Akt signaling pathway might mediate Wnt5a-induced osteosarcoma cell migration.

## Materials and methods

### Cell culture

Human osteosarcoma cell line MG-63 purchased from Cells Resource Center of Shanghai Institutes for Biological Sciences, Chinese Academy of Sciences (Shanghai, China) and were cultured in Dulbecco-modified Eagle’s medium (DMEM) supplemented with 10% fetal bovine serum (FBS).

### Small interfering RNA (siRNA)

For gene knockdown, siRNA duplexes specific for Akt (Cell Signaling) were transfected into MG-63 cells by using Lipofectamine 2000 reagent (Invitrogen, Carlsbad, CA) in serum-free OPTI-MEM according to the manufacturer’s instructions. Knockdown efficiency was evaluated 48 h after transfection by measuring protein levels in cell lysates through using immunoblotting.

### Immunoblotting analysis

Subconfluent cells were washed twice with PBS, and then lysed with ice-cold RIPA lysis buffer (50 mmol/L Tris, 150 mmol/L NaCl, 1% Triton X-100, 1% sodium deoxycholate, 0.1% SDS, 1 mmol/L sodium orthovanadate, 1 mmol/L sodium fluoride, 1 mmol/L EDTA, 1 mmol/L PMSF, and 1% cocktail of protease inhibitors) (pH7.4). The lysates were then clarified by centrifugating at 12,000 g for 20 min at 4°C. The protein extracts were separated by SDS-PAGE. The immunoblotting procedure was performed as described [16] and the following antibodies were used: mouse anti-β-actin antibody (KangChen Bio-tech, Shanghai, China), rabbit anti-Akt antibody, rabbit anti-phospho-Akt (p-Ser473) antibody, rabbit anti-PI3K p85 antibody, rabbit anti-phospho-PI3K p85 (Tyr458) (Cell Signaling Technology, Danvers, MA), mouse anti-β-catenin antibody, rabbit anti-phospho-β-catenin (p-Ser33) antibody (Santa Cruz Biotechnology, Santa Cruz, CA). Protein bands were detected by incubating with horseradish peroxidase-conjugated antibodies (Santa Cruz Biotechnology, Santa Cruz, CA) and visualized with ECL reagent (Thermo Scientific, Rockford, IL).

### Wound healing assay

MG-63 cells were plated onto 96-well cell culture clusters (Costar) and grown to confluence, and then serum-starved for 24 h. The monolayer cells were scratched manually with a plastic pipette tip, and after two washes with PBS, the wounded cellular monolayer was allowed to heal for 10 h in DMEM containing 100 ng/ml recombinant Wnt5a (rWnt5a) (R&D Systems). Photographs of central wound edges per condition were taken at time 0 and at the indicated time points using digital camera (Nikon, Tokyo, Japan).

### Cell migration assays

Cell migration was assessed in a modified Boyden chamber (Costar), in which two chambers were separated by a polycarbonate membrane (pore diameter, 8.0 μm). MG-63 cells were grown to subconfluence in tissue culture plates and then detached; thereafter, they were centrifuged and rendered into single cell suspensions in serum-free culture medium supplemented with 5 μg/mL BSA. The suspensions containing 5 × 10^4^ cells were added to wells with a membrane placed in the bottom. Medium containing indicated Wnt5a was added to the upper and lower compartment of the Boyden chamber. The cells were allowed to migrate for the indicated periods of time at 37°C in this assay. Thereafter, the medium was discarded, stationary cells were removed with a cotton-tipped applicator and the membranes were cut out of the chamber and stained with 0.5% crystal violet. The response was evaluated in a light microscope by counting the number of cells that had migrated into the membrane.

### Statistical analysis

All experiments here were repeated at least three times, with independent treatments, each of which showed essentially the same results. The data were analyzed using Student’s *t*-test by SPSS statistical software package. All the results were expressed as mean ± SD. For all analyses a two-sided *p* value of less than 0.05 was deemed statistically significant. This study had been approved by institutional ethics committee of Changzhou No. 1 People’s Hospital.

## Results

### Wnt5a stimulates osteosarcoma cell migration in vitro

**Figure 1:**
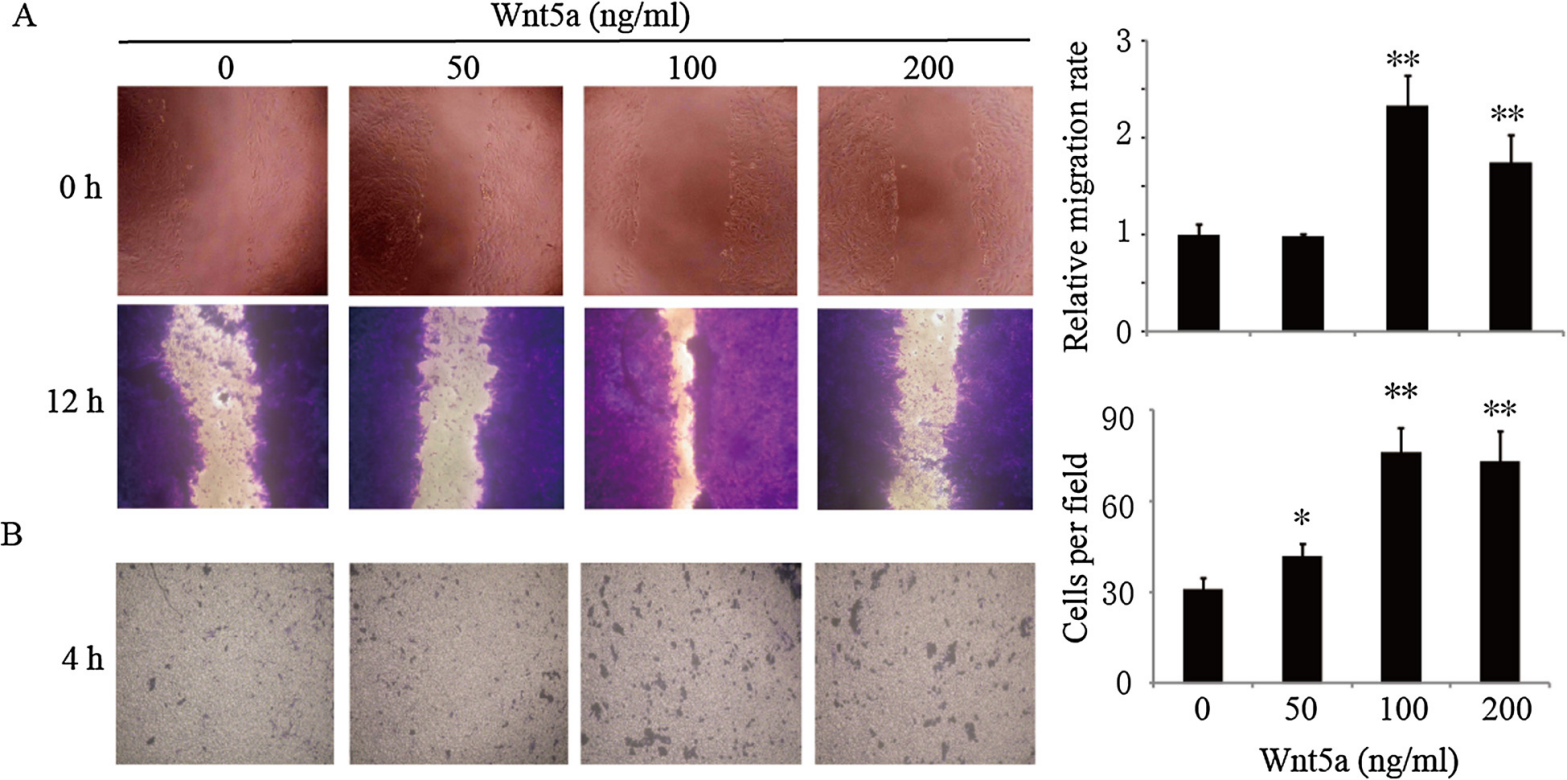
**Effect of Wnt5a on the migration of osteosarcoma cells.** Relative cell migration rate was determined by using wound healing assay **(A)** and Boyden chamber assays **(B)** in MG-63 cells incubated in the absence (0 ng/ml) or presence of 50, 100, and 200 ng/ml Wnt5a for 10 h. *, **: *p* < 0.05, *p* < 0.01 in the cultures with Wnt5a relative to the cultures without Wnt5a, respectively. Data were presented as mean ± SD of 5 determinations.

To assess the effect of Wnt5a on osteosarcoma cell migration, we treated MG-63 cells with different doses of recombinant Wnt5a (rWnt5a), and measured the migration rate by wound healing assays and Boyden chamber assays. We found that Wnt5a had a potent stimulatory effect on MG-63 cell migration (Figure 1A, 1B). An approximately 2-fold increase in cell migration was observed in cells treated with 100 ng/ml rWnt5a (Figure 1A, 1B). Nevertheless, low concentration of Wnt5a (50 ng/ml) has no stimulative effect on MG-63 cell migration (Figure 1A). Accordingly, 100 ng/ml rWnt5a was used for the remaining studies hereafter to identify the mechanism that accounts for the changes in the migration of MG-63 cells.

### Wnt5a induces PI3K and Akt phosphorylations

**Figure 2:**
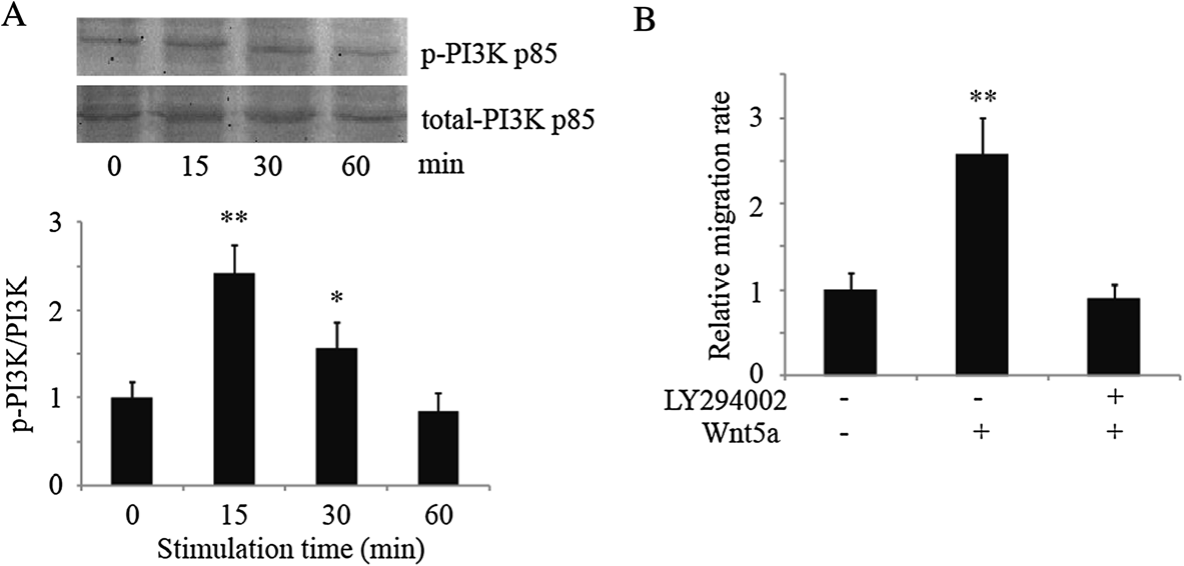
**Wnt5a induces PI3K activation of osteosarcoma cells. (A)** Human osteosarcoma cells MG-63, serum-deprived for 24 h, were untreated or treated with 100 ng/ml of Wnt5a and harvested at 15 min, 30 min, and 1 h after the start of treatment for SDS-PAGE and immunoblot analysis with antibodies to p-PI3K p85 (p-Tyr458) and total PI3K p85. p-PI3K/total PI3K ratios were indicated at each time. Data were presented as mean ± SD of 3 determinations. *, **: *p* < 0.05, *p* < 0.01 in the cultures with Wnt5a relative to the cultures without Wnt5a. **(B)** Serum-deprived MG-63 cells were pre-treated with 20 μM LY294002 for 1 h. Relative cell migration rate was determined by using wound healing assay in MG-63 cells incubated in the absence or presence of 100 ng/ml Wnt5a for 10 h. ** *p* < 0.01 in the cultures with Wnt5a relative to the cultures without Wnt5a. Data were presented as mean ± SD of 5 determinations.

Wnt5a-triggered signals in human osteosarcoma cells have remained completely unknown. To address the question, we first tried to identify the downstream signals triggered by Wnt5a in MG-63 cells. We first detected the phosphorylated-PI3K p85 (p-Tyr458), which represents the PI3K activation state. Human osteosarcoma cells, serum-starved for 24 h, were treated with 100 ng/ml of rWnt5a. The cells were harvested at 15 min, 30 min and 1 h after the start of Wnt5a treatment, followed by SDS-PAGE and immunoblot analyses. PI3K showed visible signs of basal phosphorylation and elevated phosphorylation at 15 min after stimulation with rWnt5a and continued to be elevated at least until 30 min after the start of treatment with Wnt5a (Figure 2A).

**Figure 3:**
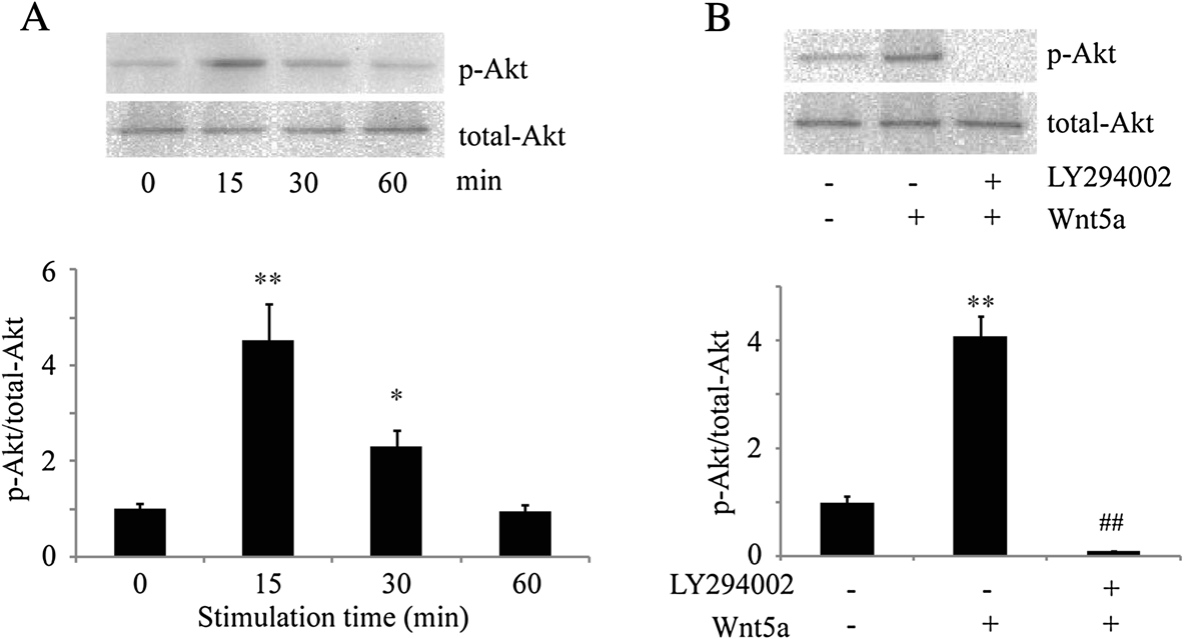
**Wnt5a promotes cell migration via Akt pathway. (A)** Human osteosarcoma cells MG-63, serum-deprived for 24 h, were untreated or treated with 100 ng/ml of Wnt5a and harvested at 15 min, 30 min, and 1 h after the start of treatment for SDS-PAGE and immunoblot analysis with antibodies to p-Akt (p-Ser473) and total Akt. p-Akt/total Akt ratios were indicated at each time. Data were presented as mean ± SD of 3 determinations. *, **: *p* < 0.05, *p* < 0.01 in the cultures with Wnt5a relative to the cultures without Wnt5a. **(B)** An equal number of MG-63 cells were pre-treated with 20 μM LY294002 (PI3K inhibitor) for 1 h, then incubated with 100 ng/ml Wnt5a for 15 min. After stimulation, cells were analyzed by immunoblotted with p-Akt (p-Ser473) or total Akt antibodies. Data were presented as mean ± SD of 3 determinations. **, ##: *p* < 0.01, *p* < 0.01, in the cultures with Wnt5a relative to the cultures without Wnt5a.

The most established activator of Akt is PI3K, therefore we sought to determine whether Akt activation was triggered by Wnt5a. The same assays were performed to detect the phosphorylated-Akt (p-Ser473), which represents the Akt activation state. Akt also showed visible signs of basal phosphorylation and elevated phosphorylation at 15 min after stimulation with rWnt5a and continued to be elevated at least until 1 h after the start of treatment with Wnt5a (Figure 3A).

### Wnt5a promotes cell migration via PI3K pathway

The finding that Wnt5a could induce PI3K/Akt phosphorylation in MG-63 cells prompted us to determine whether PI3K/Akt activation was required for Wnt5a-mediated cell migration. Wnt5a-induced cell migration was largely abolished by pre-treatment with 20 μM LY294002, the PI3K-specific inhibitor (Figure 2B), suggesting that PI3K activation is required for Wnt5a-induced MG-63 cell migration.

To demonstrate the involvement of PI3K in Wnt5a-induced activation of Akt, we tested the effect of LY294002 for Akt activation. Human osteosarcoma cells, serum-starved for 24 h and pretreated with 20 μM LY294002 for 1 h, were incubated with 100 ng/ml of Wnt5a. The cells were harvested 15 min after the start of Wnt5a treatment and the cell lysates were subjected to SDS-PAGE and immunoblot analysis. The Wnt5a-induced activation of Akt was mostly blocked by pretreatment of LY294002 (Figure 3B). These data indicate that PI3K mediates Wnt5a-induced activation of Akt.

### Akt activation regulates osteosarcoma cell migration

**Figure 4:**
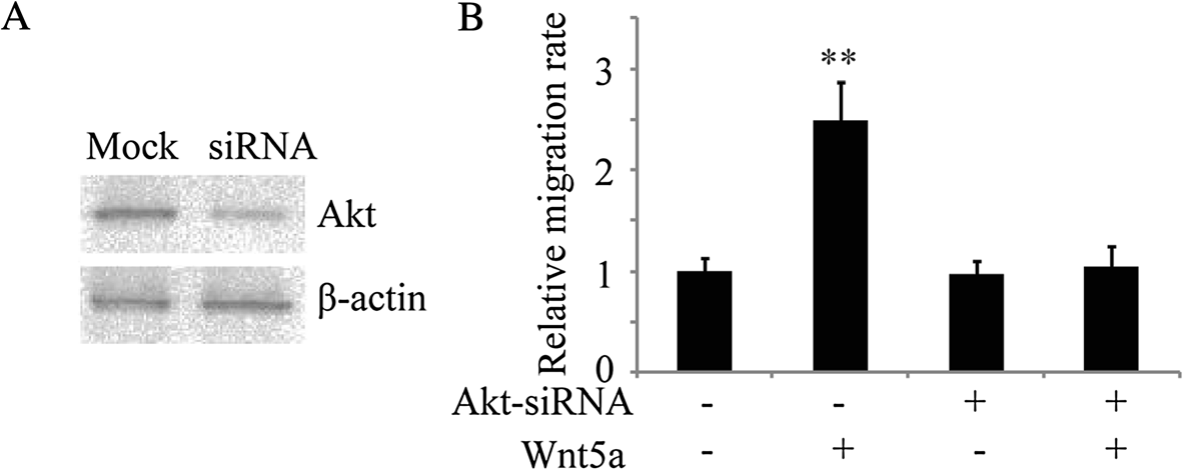
**Effect of Akt in Wnt5a-induced cell migration. (A)** Effect of Akt siRNA on the expression of Akt. MG-63 cells were transiently transfected with Akt siRNA pool, or mock for 48 h. Cells were analyzed by immunoblotted with total Akt antibody. **(B)** Effect of Akt siRNA on Wnt5a-stimulated cell migration. Cells were transiently transfected with Akt siRNA pool, and stimulated with 100 ng/ml Wnt5a or not for 10 h. Relative cell migration rate was determined by using wound healing assay. ** *p* < 0.01 in the cultures with Wnt5a relative to the cultures without Wnt5a. Data were presented as mean ± SD of 5 determinations.

To analyze the role of endogenous Akt activation on Wnt5a-induced cell migration, we knocked down Akt expression by using siRNA, which reduced the protein level of Akt by approximately 60%, as assessed by immunoblotting (Figure 4A) and significantly reduced Wnt5a-induced migration of MG-63 cells (Figure 4B). Taken together, these experiments demonstrated that Akt activation was required for Wnt5a-induced MG-63 cell migration.

### Wnt5a does not alter the total expression and phosphorylation of β-catenin

**Figure 5:**
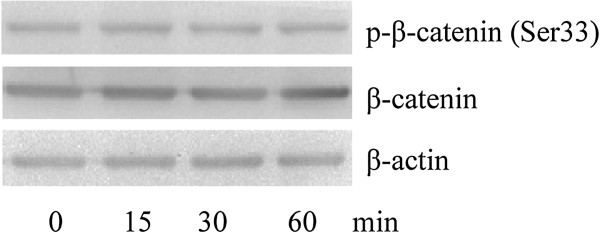
**Wnt5a does not alter the total expression and phosphorylation of β-catenin.** MG-63 were untreated or treated with 100 ng/ml of Wnt5a and harvested at 15 min, 30 min, and 1 h after the start of treatment for SDS-PAGE and immunoblot analysis with antibodies to p-β-catenin (p-Ser33) and total β-catenin. The total expression and phosphorylation of β-catenin were not been altered by Wnt5a stimulation.

Mikels and Nusse reported that purified Wnt5a inhibits Wnt3a protein-induced canonical Wnt signaling in a dose-dependent manner, not by influencing β-catenin levels but by downregulating β-catenin-induced reporter gene expression in HEK293 cells or mouse L cells [17]. To assess the effect of Wnt5a on the total expression and phosphorylation of β-catenin, we treated MG-63 cells with different doses of rWnt5a, and measured the total expression and phosphorylation by immunoblotting assays. We found that the total expression and phosphorylation of β-catenin had not been altered under Wnt5a stimulation for less than 1 hour (Figure 5).

## Discussion

Wnt5a is a prototypic ligand that activates a β-catenin independent pathway in Wnt signaling [3]. Mice with the disrupted Wnt5a gene also exhibit various developmental abnormalities such as dwarfism, facial abnormalities, shortened limbs and tails, dysmorphic ribs and vertebrae, absence of the genital tubercle, and abnormal distal lung morphogenesis, indicating that it plays a crucial role in the development of various organs [18]. Wnt5a has been demonstrated to exert differential effects on cancer development [5]. Wnt5a can promote cancer progression and metastasis in malignant melanoma, breast cancer, and gastric cancer [19-23]. However, some evidence supports the hypothesis that Wnt5a acts as a tumor suppressor in certain experimental systems [24-28]. The first observation in the present study was that Wnt5a induced the migration of MG-63 osteosarcoma cells. Our finding suggest that Wnt5a acts as a migratory stimulator in osteosarcoma cells.

Kinases show the comprehensive functions triggered by Wnt5a in a variety of cells. It has been reported that Wnt5a may promote tumor progression through inducing actin reorganization and increasing cell motility via activating the protein kinase C (PKC) [19,29]. Wnt5a activates extracellular regulated protein kinase (ERK) in endothelial cells [30]. Wnt5a prevents apoptosis caused by serum-deprivation in osteoblasts by activating both Akt and ERK [12], and promotes cell adhesion in mammary epithelial cells in a PI3K/Akt-dependent manner [31]. Furthermore, Wnt5a triggers Akt phosphorylation via PI3K, but not ERK or PKC phosphorylation in human dermal fibroblasts [14]. These tanglesome viewpoints of kinases in Wnt5a signaling indicate that phosphorylated signaling molecules triggered by Wnt5a in these cases determine the diverse roles of these kinases. In this study, we demonstrated that Wnt5a promoted MG-63 osteosarcoma cell migration by activating PI3K/Akt. We found that Wnt5a induced PI3K (p-Tyr458) and Akt phosphorylation (p-Ser473) rapidly and transiently in MG-63 cells. Blocking Akt signaling by chemical inhibition of PI3K or Akt siRNA completely abolished Wnt5a-induced cell migration, indicating that PI3K/Akt activation participated in the regulation of the MG-63 cell migration.

The activation of PI3K/Akt signaling is thought to play a central role in cell viability-promoting phenotypes such as inhibition of cell death and cell cycle progression [14]. It has been shown that the PI3K/Akt signaling could be activated not only by Wnt5a but also by Wnt3a. Wnt3a prevents the serum deprivation-triggered apoptosis of osteoblasts by activating the canonical β-catenin pathway, ERK-, and/or PI3K-mediated uncharacterized pathways [12]. In this study, we have showed that Wnt5a promotes cell migration of human osteosarcoma cells in the PI3K/Akt-dependent manner. These results indicated that PI3K/Akt played crucial and complicated roles in canonical and non-canonical Wnt signalings in various cell and tissue types. Besides mentions above, PI3K/Akt signaling presents wide crosstalks referred to instances in which one or more components of one signal transduction pathway affect another. High-mobility group box 1 (HMGB1) activates the FAK/PI3K/mTOR signaling cascade and regulates tumor-associated cell migration through the interaction with BTB domain [32]. CXCR4 drives the metastatic phenotype in breast cancer through induction of CXCR2, and activation of MEK and PI3K pathways [33]. Akt signaling is involved in fucoidan-induced inhibition of growth and migration of human bladder cancer cells [34]. These studies provide us a complicated and crossed network around the PI3K/Akt signaling.

In summary, we present the first direct evidence here that Wnt5a promotes osteosarcoma cell migration via PI3K/Akt signaling. These findings elucidate a molecular pathway linking Wnt5a signaling to PI3K/Akt in cell motility. This result will contribute to further understanding of biological roles of Wnt5a/PI3K/Akt in cell migration of osteosarcoma and other cancers.

## Competing interest

The author declares that they have no competing interest.

## Authors’ contributions

AZ and SH participated in the design of this study, and they both carried out the study. XS collected important background information, together with LD, and performed the statistical analysis. XB and NW participated in the design and helped to write the manuscript. All authors read and approved the final manuscript.
